# Characterization of Normal and Degenerative Discovertebral Complexes Using Qualitative and Quantitative Magnetic Resonance Imaging at 4.7T: Longitudinal Evaluation of Immature and Mature Rats

**DOI:** 10.3390/bioengineering12020141

**Published:** 2025-01-31

**Authors:** Benjamin Dallaudière, Emeline J. Ribot, Aurélien J. Trotier, Laurence Dallet, Olivier Thibaudeau, Sylvain Miraux, Olivier Hauger

**Affiliations:** 1 Centre de Résonance Magnétique des Systèmes Biologiques, UMR 5536, CNRS. Université de Bordeaux, 146 rue Léo Saignat, 33076 Bordeaux, France; emeline.ribot@rmsb.u-bordeaux.fr (E.J.R.); aurelien.trotier@rmsb.u-bordeaux.fr (A.J.T.); laurence.dallet@rmsb.u-bordeaux.fr (L.D.); sylvain.miraux@rmsb.u-bordeaux.fr (S.M.);; 2 Département d'Imagerie Musculo-squelettique, Centre Hospitalier Universitaire Pellegrin, Place Amélie Léon Rabat, 33000 Bordeaux, France; 3 Centre d'Imagerie Ostéo-articulaire, Clinique du Sport de Bordeaux-Mérignac 2, rue Négrevergne, 33700 Mérignac, France; 4 Inserm UMS 34 Claude Bernard, Université de Paris Cité / UFR de Médecine Site Bichat, 16, rue Henri Huchard—BP 416, 75870 Paris Cedex 1, France; olivier.thibaudeau@inserm.fr

**Keywords:** disc, UTE, MRI, anatomy, endplate, mapping, T2, T1

## Abstract

Purpose: We assessed the feasibility of qualitative, semiquantitative, and multiparametric quantitative magnetic resonance imaging (MRI) using a three-dimensional (3D) ultrashort echo time (3D-UTE) sequence together with 2D-T2 and 3D-T1 mapping sequences to evaluate normal and pathological discovertebral complexes (DVCs). We assessed the disc (nucleus pulposus [NP] and annulus fibrosus [AF]), vertebral endplate (cartilage endplate [CEP] and growth plate [GP]), and subchondral bone (SB) using a rat model of degenerative disc disease (DDD). We also assessed whether this complete MRI cartography can improve the monitoring of DDD. Methods: DDD was induced by percutaneous disc trituration and collagenase injection of the tail. Then, the animals were imaged at 4.7T. The adjacent disc served as the control. The MRI protocol was performed at baseline and each week (W) postoperatively for 2 weeks. Visual analysis and signal intensity measurements from the 3D-UTE images, as well as T2 and T1 measurements, were carried out in all DVC portions. Histological analysis with hematoxylin–eosin and Masson trichrome staining was performed following euthanization of the rats at 2 weeks and the results were compared to the MRI findings. Results: Complete qualitative identification of the normal zonal anatomy of the DVC, including the AF, CEP, and GP, was achieved using the 3D-UTE sequence. Quantitative measurements of the signal-to-noise ratio in the AF and NP enabled healthy DVCs to be distinguished from surgery-induced DDD, based on an increase in these values post-surgery. The 2D-T2 mapping results showed a significant increase in the T2 values of the AF and a decrease in the values of the NP between the baseline and W1 and W2 postoperatively (*p* < 0.001). In the 3D-T1 mapping, there was a significant decrease in the T1 values of the AF and NP between baseline and W1 and W2 postoperatively in immature rats (*p* < 0.01). This variation in T1 and T2 over time was consistent with the results of the 3D-UTE sequence. Conclusions: Use of the 3D-UTE sequence enabled a complete, robust, and reproducible visualization of DVC anatomy in both immature and mature rats under both normal and pathological conditions. The findings were supported quantitatively by the T2 and T1 mapping sequences and histologically. This sequence is therefore of prime interest in spinal imaging and should be regularly be performed.

## 1. Introduction

Imaging of the discovertebral complex (DVC) is challenging due to the high diversity of its tissues, which include the highly hydrated nucleus pulposus (NP) and cartilaginous growth plate (GP), as well as the collagenous fibrous annulus fibrosus (AF) and porous cancellous cartilage endplate (CEP) [1,2,3,4]. Conventional magnetic resonance imaging (MRI) sequences do not detect all components, which has limited the precise imaging of the DVC [5] and the early MRI assessment of its pathologies. This is particularly true regarding mechanical changes related to degenerative disc disease (DDD). However, DVC and DDD have been extensively characterized in terms of their gross morphology, biochemistry, and histology. Noninvasive studies of normal DVC and degenerative changes in this structure have been performed in humans using the T1 or T2 relaxation time, ultrashort-TE (UTE) sequences, quantitative high-resolution spectroscopy, diffusion tensor imaging, and sodium MRI. While the results have been promising, a histologic correlation of the results with histological data has not been possible [6,7,8,9,10,11,12,13]. Quantitative MRI analysis using an automatic voxel-based relaxometry approach with deep-learning-based segmentation has also been used to map the DVC and to quantify and characterize DDD in humans [14,15].

Very few studies have focused on the changes in the DVC that occur with aging. Two previous studies examined gene expression in the NP over time in both normal and pathological animal models and reported significant quantitative differences that suggested different pathways in the process of disc degeneration, depending on the nature of the underlying disease [16,17]. Another study used MRI quantitative cartography and the apparent diffusion coefficient (ADC) to determine the quality of the nutritional supply of the DVC, particularly the NP, at various ages [18]. Recently, Dallaudière et al. [19] reported that 3D-UTE sequences enable a visualization of the complete DVC anatomy in a rat model. The authors also provided a rat model with histological correlation and showed that, in DDD, early stages could be differentiated from chronic stages in immature rats. However, that study lacked the quantitative information needed to validate the qualitative data, and mature rats were not included. Thus, to fill these gaps and to build a robust protocol for routine imaging, we conducted a more comprehensive study.

## 2. Materials and Methods

### 2.1. Animal Model

The procedures and animal care complied with the “principles of animal care” of the European Union. Animal experimentation was performed under the authorization of the Ministry of Agriculture.

This study included eight female immunocompetent Sprague Dawley rats (Janvier Labs, France): four 8-week-old immature rats (mean weight = 184 ± 21 g) and four 14-week-old mature rats (mean weight = 255.4 ± 4 g). Indeed, precise anatomy of the DVC, using immature and mature rats gives us a better understanding of its physiological changes with aging, notably the two portions of the endplate with growth [19]. They were housed in groups of two in cages in a conventional animal housing facility with a 12 h light/12 h dark cycle and a temperature of 20 ± 2 °C. Mature rats were allowed to acclimate to the housing facility for 5 days or for 6–8 weeks to reach a mature age of 14 weeks. DDD in the DVC was induced through a mechanical/chemical method using intradiscal trituration [10] with an 18-gauge needle and a single intradiscal injection of type 1 collagenase (Gibco™; 20% dissolved in 100 µL saline solution) by the same operator. The mechanical/chemical method is considered simpler than, but just as efficient as, surgical or infectious models and results in an animal model of DDD as early as 3 days after treatment [20,21,22,23]. The rats were clinically observed every 2 days, monitoring for weight loss and general behavior.

### 2.2. Imaging

In vivo imaging of the DVC was performed at baseline (day 0 [D0], n = 8 animals/16 DVC) and weekly (W) for 2 weeks after DDD induction using a 4.7T Bruker Biospec system (Ettlingen, Germany) equipped with a gradient system capable of a maximum strength of 660 mT/m and a rise time of 110 µs. A volume resonator (75.4 mm inner diameter, active length 70 mm) operating in quadrature mode was used for transmission, and a four-element (2 × 2) phased array surface coil (outer dimensions of one element: 12 × 16 mm^2^; total outer dimensions 26 × 21 mm^2^) was used for signal reception. This approach increased the resolution and provided a better contrast-to-noise ratio (CNR) during examination of the rat tails.

#### Imaging Protocol

Once fully anesthetized, the animals were placed in a prone position in the MRI unit, with the proximal portion of the tail located at the center of the MR coil. Respiratory activity was monitored during all MR acquisitions. The MR protocol included an anatomical 3D-UTE sequence, a parametric 2D-T2 mapping sequence, and a 3D-T1 mapping sequence with the following parameters:

- 3D-UTE acquisition: TR/TE: 11.863/0.031 ms, FOV 17 × 17 × 17 mm, matrix: 128 × 128 × 128, spatial resolution = 0.133 mm isotropic, number of excitations: 3, projections = 51,360, bandwidth = 100 kHz, flip angle = 10.0°, hard excitation pulse of 0.05 ms, fat saturation module: pulse (bandwidth 701.19 Hz, duration 7.6 ms, angle 90°), total acquisition time 30 min 28 s.
- 2D-T2 mapping using a sagittal oriented multi-slice multi-echo spin-echo (MSME) sequence: 100 images acquired at minimum TE (3.8 ms) to 378.6 ms, TR: 8000 ms, FOV 20 × 15 mm, matrix: 128 × 96, spatial resolution = 0.156 mm isotropic, 20 slices of 0.6 mm thickness, number of excitations: 1, bandwidth = 71.4 kHz, 90° 2 ms pulse followed by a 180° 1.8 ms pulse, total acquisition time 12 min 48 s.
- 3D-T1 mapping with the MP2RAGE sequence: TI1/TI2/MP2RAGE_TR_ = 800 ms/2200 ms/6250 ms; FOV = 22.5 × 22.5 ×11.25 mm; matrix = 128 × 128 × 64; spatial resolution: 0.176 × 0.176 × 0.176mm; hyperbolic secant inversion pulse (10 ms); flip angles = 7°/7° (0.6 ms sinc10H excitation pulses); 160 echoes per GRE readout; TR/TE = 6/2.16 ms; rBW = 50 kHz; number of excitations: 4; scan time: 21 min 15 s [24,25].

The average total exam time, including optimal installation, was ~65 min. At the end of the longitudinal follow-up, tail specimens were removed from the animal, incubated with 4% paraformaldehyde overnight for tissue fixation, and then placed in a 15 mL tube containing phosphate-buffered saline.

### 2.3. Image and Parametric Map Reconstruction

To obtain T2 maps, a pixel-wise curve fitting of the signal from each echo was performed using the following equation: *S*(*t*) = (*S* .*e*xp(−t/T2)) + 2.*L*.*σ*^2^, with L the amount of array within the receive coil (here 4) and *σ* the noise.

To build T1 maps, the conventional equations from Marques JP et al. [26]. were used. Briefly, the two images (GRE1 and GRE2) were combined using the following equation:

$MP2RAGE=R\left(\frac{GRE_{1}^{*}GRE_{2}}{{\left|GRE_{1}\right|}^{2}+{\left|GRE_{2}\right|}^{2}}\right)$. Then, the MP2RAGE signal of each voxel was used to build the T1 map through a look-up-table that was simulated using the main sequence parameters [26].

### 2.4. Image Analysis

Images were analyzed using MATLAB-based software (MATLAB^®^, R2024A). Qualitative analysis of the DVC, quantitative signal intensity measurements (from the in vivo 3D-UTE images), and T2 and T1 quantification were performed by a musculoskeletal radiologist and a physicist (BD and ER) with 10 years of experience each. For quantitative analysis, two regions of interest (ROIs) were drawn per animal on the sagittal images within the healthy and the pathological DVC to delineate the NP, AF, cartilage endplate (CEP), growth plate (GP), and sub-chondral bone (SB) of normal and degenerative DVCs. Also, an ROI in the background noise was drawn to measure signal-to-noise ratio (SNR) on the UTE images. The results are expressed as the SNR (mean signal within a ROI divided by the standard deviation of the noise) and the CNR (difference in SNR between two structures of interest) for 3D-UTE, as the T2 values ± standard deviation (SD) for the T2 mapping, and as the T1 values ± SD for the T1 mapping. The SNR was determined by dividing the mean signal value over an ROI by the mean magnitude of the noise divided by 2.74. The latter value is used to correct the Rayleigh distribution of noise for a four-element phased array coil [27]. The CNR was determined by subtracting the SNR of two neighboring ROIs (AF/NP, AF/CEP, CEP/GP, and GP/SB). The T1 and T2 values were measured as the mean value over an ROI.

### 2.5. DVC Pathology

After removal of the tail, spinal segments were decalcified, cut in the mid-sagittal plane, placed in 75% ethanol, and embedded in paraffin. Then they were cut in 5 µm slices and stained with hematoxylin–eosin (H&E) and Masson trichrome (MT) stain. The sagittal plane served as the reference, as it allowed optimal correlation with the histology. All of the DVC (NP, AF, CEP, GP, and SB) was assessed. The pathology exam considered the disorganization of the NP, fissuring of the AF, a rift in the EP, and SB edema and neovascularization [3,4].

### 2.6. Statistical Analysis

Statistical analysis was performed using R-software (R version 4.1.2). Aberrant values were excluded from the statistics in comparisons of DDD and healthy discs. Comparisons were also made between the values from immature and mature rats, in which case no values were excluded. After validation of the normality (Shapiro test) and the homogeneity of variance (Bartlett test), a parametric T test or a nonparametric test (Mann–Whitney test) was used to compare the in vivo data obtained from all sequences at the different locations of the DVC at the different time points. A *p* value < 0.05 was considered to indicate statistical significance.

## 3. Results

At day 0, all animals were asymptomatic without evidence of pain. All rats survived the intradiscal procedure. The qualitative and quantitative measurements of the healthy and pathological groups are summarized in Table 1, Table 2, Table 3, Table 4 and Table 5.

### 3.1. Image Analysis

#### 3.1.1. Qualitative Analysis of the Normal DVC (Table 1 and Table 2)

**Figure 1:**
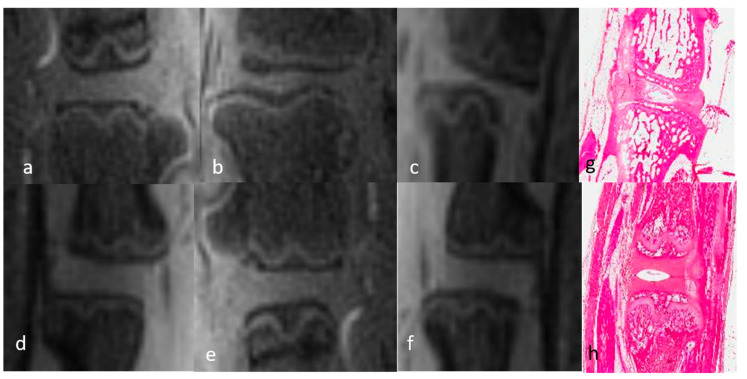
Sagittal 3D-UTE-weighted acquisition sequences of the discovertebral complex (DVC) of immature rats during in vivo assessment at day 0 (D0) and week (W)1 and W2. In the 3D-UTE images of a rat model of degenerative disc disease (DDD) (**a**–**c**), both the NP and the AF appear as uniformly hyper-intense signals at W1. The CEP and GP are unchanged compared to the healthy DVC. At W2, the entire disc appears as a heterogenous high-intensity signal, less bright than at W1, suggesting a decrease in the mechanical discitis. CEP, GP, and SB signals are unchanged. In the 3D-UTE sequence of the normal DVC (**d**–**f**), the NP appears as a homogeneous low-intensity signal, and the AF as a uniform high-intensity signal. The GP can be clearly identified in its entirety, from the anterior to posterior vertebrae cortex, as a bright continuous line between the CEP and the SB, with a homogeneous low-intensity signal. Upper DVC: normal DVC at day (D) 0 (before DDD induction) and DDD at week (W)1 and W2. Lower DVC: normal control DVC at D0, W1, and W2. DVC at D0 (**h**) and DDD histology at W2 (**g**) in immature rat (H&E staining), showing the global disorganization of the NP and AF. The vertebral endplate anatomy is unchanged.

**Figure 2:**
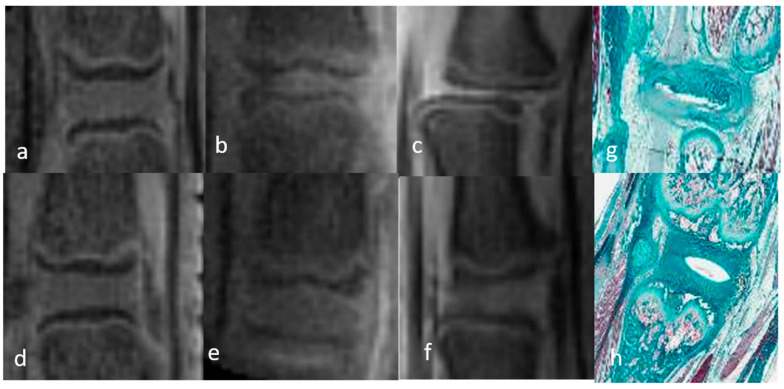
Sagittal 3D-UTE-weighted acquisition sequences of the discovertebral complex (DVC) of a mature rat during in vivo assessment at day 0 (D0) and week (W)1 and W2. The MRI findings in mature rats were the same, with the exception of the thickness of the CEP, which was greater, and GP, which was nonlinear in immature rats (**a**–**f**). Upper DVC: normal DVC at day (D) 0 (before DDD induction) and DDD at week (W)1 and W2. Lower DVC: normal control DVC at D0, W1, and W2. DVC at D0 (**h**) and DDD histology at W2 (**g**) in mature rat (Masson trichrome staining), showing the global disorganization of the NP and AF. The vertebral endplate anatomy is unchanged.

The 3D-UTE sequences allowed visualization of the complete DVC anatomy in both groups of rats. The NP appeared as a homogeneous low-intensity signal and the AF as a uniform high-intensity signal due to its short T2 components. The high signal intensity of the AF allowed its differentiation from the underlying CEP, which was of low intensity. The GP could be clearly identified in its entirety, from the anterior to the posterior vertebral cortex, as a bright continuous line between the CEP and the SB characterized by a homogeneous low-intensity signal due to the fat saturation module. The only difference in the DVCs of mature and immature rats was in the thickness of the GP and CEP, which was greater in immature rats (Figure 1 and Figure 2). The T2 and T1 mapping sequences were not analyzed qualitatively.

**Table 1:** Main qualitative results of a visual analysis of the normal discovertebral complex (DVC) in immature and mature rats as determined from the in-vivo 3-dimensional ultrashort echo-time (3D-UTE) images.

Normal DVC	3D UTE	
	Mature	Immature
NP	+++	+++
AF	+++	+++
CEP	+++	+++
GP	+++	+++
SB	++	++

AF: annulus fibrosus; NP: nucleus pulposus; CEP: cartilage endplate, GP: growth plate, SB: subchondral bone. -: not visible or analyzable; +: visible but no analyzable; ++: visible and partially analyzable; +++: visible and totally analyzable.

**Table 2:** Main qualitative results of a visual analysis of the different parts of the DVC in normal immature (IR) and mature (MR) rats as determined from the in-vivo 3D UTE images at baseline.

3D UTE Images	DO IR	DO MR
NP	Uniformly hyposignal	Uniformly hyposignal
AF	Uniformly hypersignal	Uniformly hypersignal
CEP	Uniformly thick asignal	Uniformly thin signal
GP	Uniformly thick hypersignal	Uniformly thin hypersignal
SB	Uniformly hyposignal	Uniformly hyposignal

AF: annulus fibrosus; NP: nucleus pulposus; CEP: cartilage endplate, GP: growth plate, SB: subchondral bone.

#### 3.1.2. Qualitative Analysis of the DVC in DDD Rats (Table 3)

In the 3D-UTE images obtained at W1 from rats with DDD, both the NP and AF appeared as uniformly hyper-intense signals, consistent with the presence of mechanical discitis. The CEP and GP were unchanged compared to the normal DVC. The high signal of the entire disc allowed a clear identification of the CEP, which showed no abnormalities and particularly no rifts. No edema was observed in the SB. At W2, the entire disc (NP and AF) appeared as a heterogenous high-intensity signal that was not as bright as at W1, suggesting a decrease in mechanical discitis. The CEP, GP, and SB signals were unchanged (Figure 1 and Figure 2). The T2 and T1 mapping sequences were not qualitatively analyzed.

**Table 3:** Main qualitative results of a visual analysis of the pathologic DVC in immature (IR) and mature (MR) rats as determined from the in vivo 3D UTE images during follow-up from baseline to week 2.

		DO IR	DO MR	W1 IR	W1 MR	W2 IR	W2 MR
3D UTE	NP	Uniformly isosignal	Uniformly isosignal	Heterogeneity hypersignal	Heterogeneity hypersignal	Heterogeneity hypersignal	Heterogeneity hypersignal
	AF	Uniformly hypersignal	Uniformly hypersignal	Heterogeneity hypersignal	Heterogeneity hypersignal	Heterogeneity hypersignal	Heterogeneity hypersignal

AF: annulus fibrosus; NP: nucleus pulposus.

#### 3.1.3. Quantitative Analysis of the Normal DVC (Table 4)

Among healthy rats, the UTE SNR values of the AF and NP of the mature rats increased over the duration of the study (NP UTE was significantly different between baseline and W2, *p* < 0.03) but not significantly for AF, whereas in the immature rats they remained stable. The UTE CNR between the AF and NP also did not significantly vary over time in either group.

**Figure 3:**
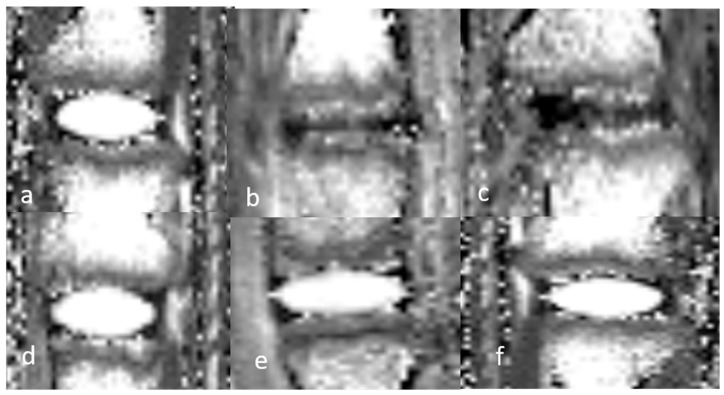
Sagittal 2D T2 mapping acquisition sequences of the discovertebral complex (DVC) of immature rats during in vivo assessment at day 0 (D0) and week (W)1 and W2 for quantitative cartography. Upper DVC (**a**–**c**): normal DVC at day (D) 0 (before DDD induction) and DDD at week (W)1 and W2. Lower DVC (**d**–**f**): normal control DVC at D0, W1, and W2.

**Figure 4:**
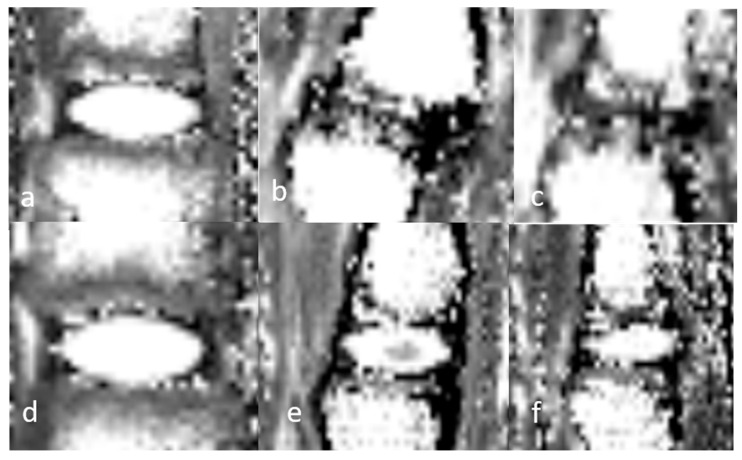
Sagittal 2D T2 mapping acquisition sequences of the discovertebral complex (DVC) of mature rats during in vivo assessment at day 0 (D0) and week (W)1 and W2 for quantitative cartography. Upper DVC (**a**–**c**): normal DVC at day (D) 0 (before DDD induction) and DDD at week (W)1 and W2. Lower DVC (**d**–**f**): normal control DVC at D0, W1, and W2.

The T2 values of the AF and NP did not significantly change over time in either group. While the AF values of mature rats slightly decreased from D0 to W1 and slightly increased from W1 to W2, the differences were not significant. Baseline values in healthy rats were around 6 ± 2.7 ms for the AF, 105 ± 12.2 ms for the NP, 28.2 ± 5.6 ms for the CEP, and 96.1 ± 17.4 ms for the SB (Figure 3 and Figure 4).

**Figure 5:**
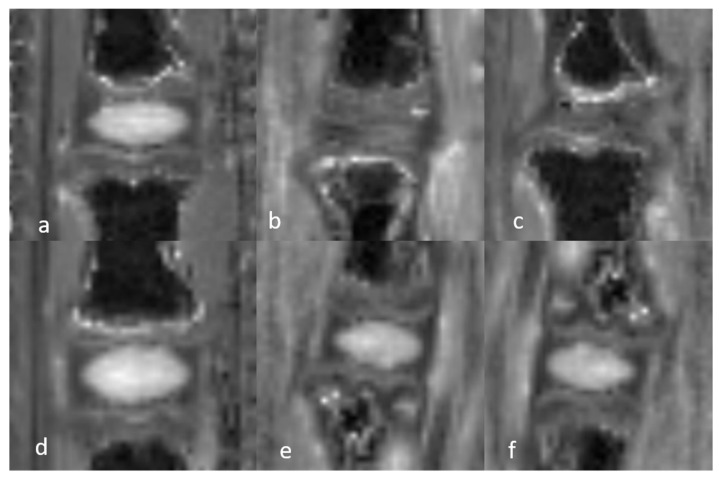
Sagittal 3D T1 mapping acquisition sequences of the discovertebral complex (DVC) of immature rats during in vivo assessment at day 0 (D0) and week (W)1 and W2 for quantitative cartography. Upper DVC (**a**–**c**): normal DVC at day (D) 0 (before DDD induction) and DDD at week (W)1 and W2. Lower DVC (**d**–**f**): normal control DVC at D0, W1, and W2.

**Figure 6:**
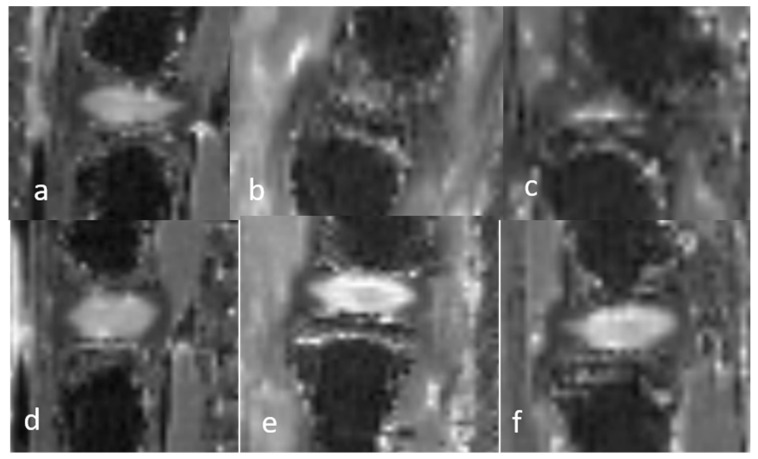
Sagittal 3D T1 mapping acquisition sequences of the discovertebral complex (DVC) of mature rats during in vivo assessment at day 0 (D0) and week (W)1 and W2 for quantitative cartography. Upper DVC (**a**–**c**): normal DVC at day (D) 0 (before DDD induction) and DDD at week (W)1 and W2. Lower DVC (**d**–**f**): normal control DVC at D0, W1, and W2.

The T1 values of the AF and NP also did not significantly differ over time in either group. Baseline normal values were 803 ± 100 ms for the AF, 1810 ± 241.8 ms for the NP, and 568 ± 48.6 ms for the SB (Figs. 2, 3, 5 and 6). Because the GP in the T2 maps and the CEP and GP on the T1 maps could not be depicted, quantitative measurements of these structures were not possible (noted “XX” in the tables).

**Table 4:** Main semiquantitative and quantitative results: signal-to-noise ratio (SNR) and contrast-to-noise ratio (CNR) evolution of the annulus fibrosus (AF) and nucleus pulposus (NP) of the healthy (h) and pathological (p) rat disc as determined from the 3D-UTE images, with the T2 values (ms) and T1 values (ms) of the rat disc calculated from the T2 mapping image, and from the MP2RAGE images. Measurements were performed on eight rats at day 0 (D0) and at week (W)1 and W2 in mature (MR) and immature rats (IR).

	3D UTE SNR		3D UTE SNR		3D UTE CNR		T2 (ms)		T2 (ms)		T1 (ms)		T1 (ms)	
	AF		NP		AF vs. NP		AF		NP		AF		NP	
	MR	IR	MR	IR	MR	IR	MR	IR	MR	IR	MR	IR	MR	IR
DO	16.8 ± 6	19.8 ± 4.3	11.5 ± 3	14.5 ± 3.5	5.3 ± 2.4	5.3 ± 2.5	6 ± 2.7	5.8 ± 2.2	104.9 ± 12.2	119 ± 15.1	803 ± 100	835 ± 96	1810 ± 242	1894 ± 187
W1h	25.7 ± 13.1	19.6 ± 4.3	20.5 ± 9.4	12.1 ± 2.7	5.2 ± 2.4	7.5 ± 4.4	3.6 ± 2.4	6.1 ± 2.6	107.6 ± 19.8	115.9 ± 26.1	804 ± 72	878 ± 102	1962 ± 149	2086 ± 294
W1p	45.6 ± 21.8	27.7 ± 6.1	35.7 ± 15.9	18 ± 1.8	9 ± 4.2	9.6 ± 4.6	20.7 ± 6.4	20.1 ± 5.2	51.8 ± 18	50.1 ± 7.5	797 ± 489	804 ± 219	1712 ± 136	1608 ± 257
W2h	37.9 ± 20	19.3 ± 4.7	30.7 ± 4.4	15.7 ± 3.9	7.2 ± 7.2	3.7 ± 4.4	4.3 ± 2.2	5.5 ± 2.7	102.8 ± 12.5	127.6 ± 21.5	837 ± 58	808 ± 62	2039 ± 55	1941 ± 72
W2p	57.1 ± 28.5	28.1 ± 8.2	52.7 ± 13.9	22 ± 4	4.3 ± 0.1	6.1 ± 4.7	14.7 ± 2.7	18.6 ± 6.3	48.5 ± 10	45.4 ± 16.5	827 ± 77	712 ± 164	1558 ± 122	1268 ± 278

#### 3.1.4. Quantitative Analysis of the DVC in DDD Rats (Table 4)

Semiquantitative and quantitative analyses focused solely on the disc (AF and NP), as it was the only target of our model and the only structure that changed over time. The UTE SNR values of the NP of DDD rats significantly increased at W1 for mature rats (*p* < 0.01) and W2 in both groups (*p* < 0.01) compared to baseline. The AF UTE-SNR increased over time, but was significantly different between baseline and W1 only for mature (*p* < 0.01) In addition, the NP signal was significantly higher in mature than in immature rats at W2 (*p* = 0.007). The SNR of both the AF and NP were higher in DDD than in healthy rats at both W1 and W2, but the difference was statistically significant only in the NP of the immature rats at W1 (*p* = 0.007). The 3D-UTE CNR between the AF and NP did not significantly vary over time, in either group, although the values increased between D0 and W1.

The AF T2 values were significantly higher at W1 and W2 than at D0 in both groups (*p* < 0.001). In parallel, NP T2 values significantly decreased (*p* < 0.001). Between W1 and W2, the AF T2 values of mature rats significantly decreased (*p* = 0.02). Both in the AF and the NP, the T2 values at W1 or W2 significantly differed between healthy and DDD rats (*p* < 0.001).

An assessment of the longitudinal relaxation times showed that the AF T1 values of mature rats did not significantly vary over time, whereas they significantly decreased in immature rats, for both W1 vs. D0 (*p* = 0.009) and W1 vs. W2 (*p* = 0.01). NP T1 values significantly decreased only in immature rats at W2 vs. D0 (*p* = 0.005). In immature but not in mature rats, the T1 values of the DDD rats were shorter than those of healthy rats, with significant differences at W1 for AF (*p* = 0.03) and at W2 for NP (*p* = 0.04) (Figure 5 and Figure 6).

#### 3.1.5. Semiquantitative and Quantitative Values of the Healthy DVC (Table 5)

As our surgical model did not show any influence on any of the DVC components except the NP and AF, there was no variation in the semiquantitative and quantitative data from the CEP, GP, or SB over time. 

**Table 5:** Main semiquantitative and quantitative results: signal-to-noise ratio (SNR), T2 values (ms), and T1 values (ms) of the healthy (h) and pathological (p) DVC. Measurements were performed on eight rats at D0, W1, and W2. MR: mature rats. IR: Immature rats.

	3D UTE SNR		3D UTE SNR		3D UTE SNR		T2 (ms)		T2 (ms)		T2 (ms)		T1 (ms)		T1 (ms)		T1 (ms)	
	CEP		GP		SB		CEP		GP		SB		CEP		GP		SB	
	MR	IR	MR	IR	MR	IR	MR	IR	MR	IR	MR	IR	MR	IR	MR	IR	MR	IR
DO	7.9 ± 0.9	10.6 ± 2.3	18.5 ± 2.6	23.5 ± 3.7	17.1 ± 27	12.9 ± 2.7	28.2 ± 5.6	25.7 ± 5	XX	XX	96.1 ± 17.4	84.8 ± 14.2	XX	XX	XX	XX	568 ± 49	574 ± 79
W1h	13 ± 6.3	10.9 ± 1.8	33.5 ± 18.1	20.2 ± 3.1	19.5 ± 11.6	10.4 ± 2.2	39.1 ± 9.6	29.9 ± 6.9	XX	XX	90.6 ± 11.4	87 ± 10.8	XX	XX	XX	XX	613 ± 93	578 ± 46
W2h	18.2 ± 5.6	11.5 ± 2.2	42.7 ± 7.6	24 ± 5.1	25.7 ± 7.1	12 ± 3	38.3 ± 4.9	28 ± 3.5	XX	XX	90.8 ± 13.1	85.3 ± 5.9	XX	XX	XX	XX	590 ± 28	579 ± 46
W1p	15.8 ± 5.7	10.8 ± 1.6	36.2 ± 12.6	19.4 ± 1.2	21.6 ± 9	12 ± 1.1	33 ± 10	27.1 ± 5.6	XX	XX	84.1 ± 6.9	89.4 ± 20.6	XX	XX	XX	XX	613 ± 74	579 ± 100
W2p	15.7 ± 6.5	11.4 ± 1.7	47.6 ± 6.1	23.9 ± 7.5	27.1 ± 6.7	13 ± 2.7	31 ± 6.5	32.5 ± 12.5	XX	XX	78.3 ± 18.9	86.3 ± 21.5	XX	XX	XX	XX	607 ± 42	555 ± 62

### 3.2. Interobserver Agreement

Kappa coefficients of 0.416 and 0.633 and 0.765 were measured for the T2 values, the SNR measurements, and the T1 values, respectively. Globally, a Kappa coefficient of 0.61 was found between the two observers.

### 3.3. Histological Evaluation

The histological examination of the H&E- and MT-stained sections showed the different components of a healthy DVC, i.e., the two portions (NP and AF) of the disc, the two portions (CEP and GP) of the endplate, and the SB. At W2 after DDD induction, the histology was consistent with important NP disruption, as well as fibrillar disorganization and a subtle loss of height in the AF. The histological appearances of the EP and the SB were normal, with no evidence of CEP modification, GP rifts, or SB edema (Figure 1 and Figure 2).

## 4. Discussion

Our study shows that 3D-UTE sequences can be used to obtain a complete visualization of the DVC. Estimation of the SNR and CNR values from those sequences, as well as 3D-T1 and 2D-T2 mapping, provided a quantitative assessment of the DVC. This protocol can therefore be employed to investigate the anatomy of the DVC and to recognize early abnormalities related to mechanical discitis, as demonstrated in our rat model of DDD. The results obtained with this approach were confirmed histologically in immature and mature rats. To the best of our knowledge, this is the first complete qualitative and quantitative MRI mapping of the DVC in healthy and DDD immature and mature rats and the first such study to include a histological correlation of the findings.

T1 and T2 mapping have been applied to validate qualitative and semiquantitative UTE data on the anatomy of the DVC and to identify lesions at early and semi-recent stages. However, very few T1 mapping sequences have been carried out in 3D, as they are time-consuming and incompatible for scanning animals with pathologies, such as in preclinical studies. With the introduction of the “magnetization prepared 2 rapid acquisition gradient echoes” (MP2RAGE) sequence, 3D T1 maps can be generated within a reasonable scan time and their precision is higher than that of other rapid sequences, such as the variable flip angle sequence. In addition, with the acquisition of two gradient–echo (GRE) images at two different inversion times, the inhomogeneity of the received B1-field is canceled. In the T2 mapping sequences, the 2D multi-echo spin-echo sequence has the advantages of a very high precision and a reasonable scan time. The advantages of the parametric maps as used in the current context were twofold. First, they enabled an assessment of the state of the intervertebral disc (either normal or pathological), thus supporting the measurements of disc thickness on the UTE images. Second, relaxation time mapping provided an on objective analysis of the serial follow-up based on a pixel-wise quantification, thereby avoiding the need for multiple observers to analyze the images [24,25,26].

Dallaudière et al. [19] performed qualitative and semiquantitative analyses of the DVC based on a 3D-UTE sequence but their study was limited to immature rats and did not include parametric quantitative T1 and T2 measurements. The results in healthy immature rats were confirmed in the present study, which, by also analyzing the DVC in mature rats, revealed a unique difference, i.e., the thickness of the CEP and GP, between the two age groups. The DVCs of mature and immature rats could be distinguished only on the UTE sequences, as only these images depicted the GP. This result is in line with those reported by Dallaudière et al. [19] despite certain differences in the 3D-MRI sequences [19]. In the present study, the 3D-UTE sequence provided a complete and detailed assessment of the DVC anatomy. The two portions (NP and AF) of the intervertebral disc were clearly differentiated, with the NP appearing darker than the AF due to the presence of a large proportion of short T2 components. Like its discal aspect, the vertebral CEP aspect was also clearly delineated on the UTE images because the underlying GP, which corresponds to the cranio-caudal ossification front of the vertebra, appeared as a linear thin structure of high signal intensity due to the high content of cells producing hyaline cartilage [16]. The UTE sequence also distinguished the low signal intensity of the CEP from the high signal intensity of the AF.

The clear identification of the zonal anatomy of the DVC provides insights into the physiological changes that occur with aging, notably the evolution of the two portions of the endplate (CEP and GP) with growth [26,27]. In rats, the ossification nucleus is rectangular in shape. As the vertebrae grows, the CEP and GP progress superiorly and inferiorly, respectively, but as the CEP becomes uniformly (from anterior to posterior) thinner, the ossification front represented by the GP becomes uniformly thicker. This evolution differs from that in humans, in which the vertebral ossification nucleus is ovoid, such that CEP progression is not uniform but follows a V-shaped pattern, with the periphery appearing thicker at the central portion of the vertebral plateau, whereas the GP is thicker at the center of the vertebral plateau and thinner at the periphery [28,29,30].

The qualitative and semiquantitative data obtained from the UTE images were confirmed by the quantitative T1 and T2 maps, with T1 and T2 values of the disc components (AF and NP) that were stable over time in healthy rats. This allowed a calculation of the mean values for use as a reference in subsequent studies. The T2 and T1 values were similar for mature and immature rats.

In DDD rats, the qualitative analysis further demonstrated the utility of the UTE sequence in mature and immature rats. In addition to the fact that this sequence precisely revealed the disc pathology, it was the only sequence to depict the CEP, thus allowing its analysis. In the UTE sequence, the CEP appeared as a hypo-intense structure located between the hyper-intense signals of the pathological disc and the GP. The ability to analyze the CEP is of clinical importance because its rupture conditions the impairment of the vertebral body—particularly in a degenerative setting that includes Modic I-type anomalies—and thus is significantly linked to the patient’s painful symptomatology. Based on our observations in DDD rats, we hypothesized that the thickness of the CEP has a protective effect in terms of preventing GP rifts and SB abnormalities, such as those induced by Modic I-type anomalies [2]. The CEP, which consists of porous cancellous bone with cavities filled with marrow and blood vessels that supply nutrients, is an early target of DDD, with GP and SB abnormalities developing thereafter. In our series, no visual extra-discal abnormalities with endplate involvement were seen in either immature or mature rats. The qualitative data were consistent with the semiquantitative data and showed a higher SNR in DDD discs than in healthy discs (whether before surgery in DDD rats or in the healthy non-operated control discs).

The qualitative and semiquantitative data obtained from the UTE images of DDD rats were confirmed by the quantitative T1 and T2 maps, both at baseline and during monitoring. The post-procedural change in the NP was characterized by a decrease in T1 and T2, consistent with the loss of the NP’s water signal. The increase in AF T2 reflected post-procedural inflammation, which would not have been prominent on the T1 images. This coherence between the UTE signal and the T1 and T2 values was maintained during follow-up imaging. Furthermore, there was no notable difference in signal behavior between mature and immature rats, with the exception of the T1 values, which decreased more markedly in immature rats. The reason for this evolution is still under investigation.

The DDD discs were characterized by collapse and a substantial decrease in the T2 values, albeit without the possibility to distinguish early from chronic discopathy [10]. The CEP was also indistinguishable from the GP. These results are in accordance with previous data showing that conventional sequences are not able to demonstrate the early signs of vertebral endplate degeneration leading to edema and Modic I changes [31,32,33]. Clauet et al. used a T2-weighted imaging sequence to analyze the NP based on Pfirrmann’s grading, without quantitative T2 determinations, in 1-, 6-, and 30-month-old rabbits. The T2 intensity of the lumbar spine was shown to decrease as a function of age. The authors correlated this evolution to changes in tissues resulting from cellular NP modification, as measured via quantitative real-time PCR [16]. Similarly, Sowa et al. found that the T2 intensity index of the NP decreased by 25% over 120 weeks in rabbits; the results correlated with those of an analysis of gene expression in the NP [17]. In humans, Stelzeneder et al. used a 3.0T MR unit to examine 330 lumbar discs from 66 patients with lower back pain [6]. Sagittal T2-weighted fast-spin echo for morphological MRI and a multi-echo spin-echo sequence for T2 mapping were performed. In agreement with our results in DDD rats, the NP T2 values showed a stepwise decrease from Pfirrmann grade I to Pfirrmann grade IV. The posterior AF had the highest T2 values in Pfirrmann group II, while the anterior AF had relatively constant T2 values in all Pfirrmann groups. The authors showed that a standardized method of quantitative T2 relaxation time evaluation is able to characterize different degrees of disc degeneration quantitatively. Similarly, in a study of 10 asymptomatic volunteers, Noebauer-Huhmann et al. reported a moderate inverse correlation between T2 mapping and the Pfirrmann score (r = −0.62) and no correlation between sodium imaging and T2 mapping (r = 0.06) [13]. Our quantitative results are fully concordant with these animal and human results. In the T2 maps, a comparison of the AF and NP values at baseline and at W1 and W2 showed a significant decrease in the NP value and increased T2 values of AF components, regardless of the age of the rats. These significant differences were also noted in comparisons of the signal intensity from the UTE images of the NP between W1 and W2, thus demonstrating that progressive signal modifications of the pathological NP can be monitored using this sequence.

Two recent studies in humans focused on measurements of the T1 relaxation times, with promising results that included a good correlation of those measurements with DDD. The first study found that symptomatic disc herniation is characterized by significantly shorter T1 relaxation times than matched asymptomatic herniation [34]. The second study evaluated the advantage of T1 mapping in the noninvasive assessment of the composition and structural integrity of the disc matrix [35].

In the 2D-T2 mapping assessment performed in the present study, reliable measurements were obtained only from the images of the NP, AF, CEP, and SB. The T2 relaxation time had the strongest correlation with the bulk water content of the tissue; in general, the wetter the tissue, the longer the T2 relaxation time, although there are also many other relaxation mechanisms. Interestingly, the contrast was reversed compared to the 3D-UTE sequence. Moreover, unlike in the UTE images, in which the CEP portion of the EP was clearly distinguishable from the GP because of a clearly different signal, the two were not distinguishable in the T2 maps, as the low value of the CEP did not provide sufficient contrast with the GP, which was also of low value [7,24,36].

**Figure 7:**
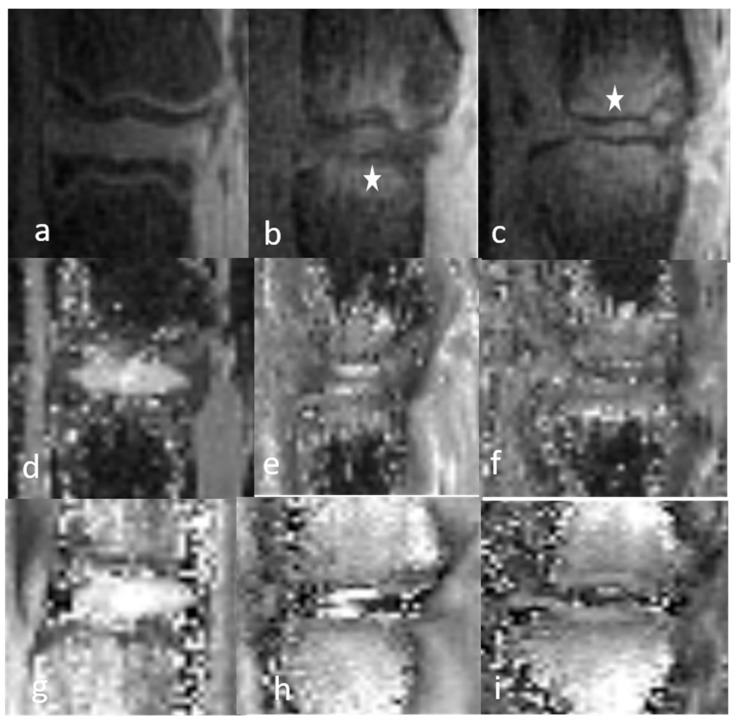
Sagittal 3D-UTE-weighted (**a**–**c**), 3D T1 mapping (**d**–**f**), 2D T2 mapping (**g**–**i**) acquisition sequence of the DVC in a mature rat with a Modic 1 lesion at W0, W1, and W2. The MRI findings of the NP and AF were the same. The CEP, GP, and SB signals indicate the pathological inflammatory condition (Modic-type lesions: white star).

The current study opens up new perspectives for spinal imaging by showing that the 3D-UTE sequence allows a complete cartography of the DVC. Our method is both robust and reproducible regardless of the intrinsic anatomy (normal or pathological) of the DVC. Spinal imaging using the 3D-UTE sequence should thus be carried out regularly. Furthermore, the semiquantitative and quantitative values of the DVC obtained in normal and DDD rats can serve as a reference in future animal studies assessing different inflammatory models. The objectives of those studies should be to evaluate the depiction ability of the 3D-UTE sequence in pathological conditions involving the CEP and GP, including those of mechanical (Modic-type lesions), inflammatory, or infectious origin, and to examine lesion progression without and under treatment. A correlation between GP signal anomalies and the lesion state of the CEP, visible only in 3D-UTE, is expected (Figure 7).

The limitations of our study should be noted as well. First, the number of animals was relatively small; however, the data collected during 2 weeks of longitudinal evaluation were sufficiently homogeneous to provide a high level of certainty regarding the results. Second, although an aggressive mechanical and chemical model of discitis was examined in this study, inflammation and DVC modifications were limited to the disc (AF and NP), whereas the underlying DVC components were unaffected. It was therefore not possible to test the ability of the 3D-UTE sequence to detect CEP and GP lesions (e.g., Modic abnormalities) and their evolution over time. Nonetheless, this does not detract from the potential applications of this sequence in normal and pathological conditions. Finally, it is unclear to what extent the results of this animal study can be extrapolated to humans. However, as the anatomy of the DVC and the types of lesions were radiologically and histologically similar, our results may shed light on the potential findings in humans.

In conclusion, the 3D-UTE sequence provides a complete, robust, and reproducible visualization of the DVC anatomy in immature and mature rats under normal and pathological (DDD) conditions. The findings were confirmed both quantitatively by T2 and T1 mapping sequences and histologically. Thus, the 3D-UTE sequence is of prime interest in spinal imaging and should be performed regularly.

## Data Availability

The data presented in this study are available on request from the corresponding author.
